# Enhanced Cytotoxicity against a Pancreatic Cancer Cell Line Combining Radiation and Gold Nanoparticles

**DOI:** 10.3390/pharmaceutics16070900

**Published:** 2024-07-05

**Authors:** Alexandra Martins, Brigida C. Ferreira, Maria Manuela Gaspar, Sandra Vieira, Joana Lopes, Ana S. Viana, António Paulo, Filipa Mendes, Maria Paula Cabral Campello, Rui Martins, Catarina Pinto Reis

**Affiliations:** 1 Departamento de Física, Faculdade de Ciências, Universidade de Lisboa, 1749-016 Lisboa, Portugal; 2 Instituto de Biofísica e Engenharia Biomédica (IBEB), Faculdade de Ciências, Universidade de Lisboa, 1749-016 Lisboa, Portugal; manuelagaspar@campus.ul.pt (M.M.G.); catarinareis@ff.ulisboa.pt (C.P.R.); 3 iMed.ULisboa, Research Institute for Medicines, Faculty of Pharmacy, Universidade de Lisboa, 1649-003 Lisboa, Portugal; joanamargaridalopes@campus.ul.pt; 4 Champalimaud Foundation, Radiotherapy, 1400-038 Lisboa, Portugal; 5 Centro de Química Estrutural, Institute of Molecular Sciences, Departamento de Química e Bioquímica, Faculdade de Ciências, Universidade de Lisboa, 1749-016 Lisboa, Portugal; apsemedo@ciencias.ulisboa.pt; 6 C2TN—Centro de Ciências e Tecnologias Nucleares, Instituto Superior Técnico, Universidade de Lisboa, 2695-066 Bobadela LRS, Portugal; apaulo@ctn.tecnico.ulisboa.pt (A.P.); fmendes@ctn.tecnico.ulisboa.pt (F.M.); pcampelo@ctn.tecnico.ulisboa.pt (M.P.C.C.); 7 DECN—Departamento de Engenharia e Ciências Nucleares, Instituto Superior Técnico, Universidade de Lisboa, 2695-066 Bobadela LRS, Portugal; 8 Centro de Estatística e Aplicações da Universidade de Lisboa, Faculdade de Ciências, Universidade de Lisboa, 1749-016 Lisboa, Portugal; rmmartins@ciencias.ulisboa.pt

**Keywords:** pancreatic cancer, megavoltage radiation therapy, gold nanoparticles, cell viability

## Abstract

The present work consisted of an exploratory study aiming to evaluate in vitro the potential of AuNPs during Radiation Therapy (RT) in human pancreatic adenocarcinoma cells. AuNPs coated with hyaluronic and oleic acids (HAOA-AuNPs) or with bombesin peptides (BBN-AuNPs) were used. AuNPs were characterized by Atomic Force Microscopy (AFM) and Dynamic Light Scattering. BxPC-3 tumor cells were irradiated with a 6 MV X-rays beam, in the absence or presence of AuNPs. AFM showed that HAOA-AuNPs and BBN-AuNPs are spherical with a mean size of 83 ± 20 nm and 49 ± 12 nm, respectively. For RT alone, a reduction in cell viability of up to 33 ± 12% was obtained compared to the control (*p* ≤ 0.0001). HAOA-AuNPs alone at 200 and 400 μM showed a reduction in cell viability of 20 ± 4% and 35 ± 4%, respectively, while for BBN-AuNPs, at 50 and 200 μM, a reduction in cell viability of 25 ± 3% and 37 ± 3% was obtained, respectively, compared to the control (*p* < 0.0001). At 72 h post-irradiation, a decrease in cell viability of 26 ± 3% and 22 ± 2% between RT + HAOA-AuNPs at 400 μM and RT + BBN-AuNPs at 50 μM, compared to RT alone, was obtained (*p* < 0.004). The combination of RT with AuNPs led to a significant decrease in cell viability compared to the control, or RT alone, thus representing an improved effect.

## 1. Introduction

In 2020, the World Health Organization estimated that 495,773 new pancreatic cancer cases were diagnosed, and 466,003 patients died from this disease [1]. As a consequence of the frequent late diagnosis of this pathology and the low effectiveness of available treatments, especially in advanced stages, the prognosis is extremely poor, with five-year survival rates below 9% [2]. Thus, new treatment approaches that address the existing challenges are largely needed, aiming to improve overall survival and patients’ quality of life. For patients with locally advanced pancreatic cancer, which represent about 30–40% of all pancreatic cancer cases, chemoradiotherapy or chemotherapy alone is recommended. However, none of the multiagent chemotherapy regimens are totally effective. Furthermore, pancreatic cancer is a deep-seated tumor surrounded by radiosensitive organs that limit the prescribed radiation dose. Despite the most recent advances in chemoradiotherapy, including cytotoxic agents and new RT techniques, pancreatic cancer is still one of the deadliest cancers [3].

Nanoparticles (NPs) with high atomic number (Z), such as gadolinium (Z = 64), hafnium (Z = 72), platinum (Z = 78), and gold (Au) (Z = 79), have been increasingly studied in the context of RT as radiation sensitizers due to their capacity to enhance radiation effects locally when introduced into tumor cells [4,5,6,7]. The basic principle is related to the high potential of these high-Z NPs to absorb low-energy X-rays, consequently leading to atomic excitation, and an increase in electrons released into the tumor tissue compared to soft tissue. Enhancement of electron release translates into a local increase in energy deposition, which causes larger damage to tumor cells compared to RT alone.

Gold NPs (AuNPs) are the most studied NPs for RT due to their high-Z, good biocompatibility, and low production cost [4,5,6,7,8]. AuNPs are colloidal or clustered particles with diameters generally ranging from 1 to 200 nm that consist of a gold core with a surface coating. Owing to their synthetic versatility, it is possible to design AuNPs with different sizes, shapes, and surface coatings [9]. The manipulation of the size and shape allows the production of AuNPs with specific chemical, electrical, and optical properties. The coating offers the possibility to control particle solubility, stability, and interaction with the biological environment [5]. These are factors of great importance as they dictate the fate of the NPs and, consequently, their accumulation in the tumor.

Size and shape are decisive factors for AuNP internalization into cells via receptor-mediated endocytosis (RME). Shape dependence is related to AuNP surface area and local curvature, both interfering with the interaction of their ligands with the cell membrane receptors [7]. Spherical AuNPs are the most common gold-based nanotherapeutics as they are simple to produce, and alteration of size and surface chemistry is easily achieved [6].

Active targeting is the most efficient method to obtain an intratumoral accumulation of AuNPs. In this strategy, the surface of the AuNPs is functionalized with peptides or antibodies that will later bind to cell surface proteins (receptors) preferentially overexpressed by cancer cells. This translates into obtaining a higher concentration of AuNPs within tumor cells compared to healthy tissues, and a reduced amount of gold needed for radiosensitization [7].

Hyaluronic acid (HA) is a natural mucopolysaccharide, highly biocompatible, that specially binds CD44, a receptor overexpressed by a wide range of tumors [10,11], including pancreatic adenocarcinomas [12,13]. Thus, conjugating AuNPs with HA may lead to a preferred accumulation of NPs into pancreatic tumor cells via RME, which in turn would translate into further harm to these cells during RT. On the other hand, bombesin (BBN) derivatives have the ability to recognize the gastrin-releasing peptide receptor (GRPR) that is overexpressed in several human tumors such as breast, prostate, and pancreatic cancer [14]. For this reason, NPs decorated with BBN peptides might promote a receptor-mediated uptake by target tumor cells to enhance the radiosensitizing effects.

The possibility of combining AuNPs with RT for cancer treatment has been widely studied over the last years through simulation, in vitro, and in vivo studies [4,5,6,7,15]. Monte Carlo simulations can be used to model the physical and physicochemical processes of radiation interactions with matter, including biological targets. This provides valuable insights into estimating AuNP-induced dose enhancement and ionizations for a range of parameters such as their size, shape, radiation source, energy, etc. Studies assessing dose enhancement by AuNPs using 80–120 kV beams obtained a dose enhancement factor near two [4]. Based solely on physical dose enhancement, radiosensitization with AuNPs is expected to be insignificant at megavoltage (MV) energies due to the minimal contribution of the photoelectric effect. However, experimental studies using MV beams with AuNPs showed an enhancement of RT effects significantly higher than those estimated by Monte Carlo simulations [5]. The discrepancies between theoretical predictions and experimental data opened the possibility of chemical and/or biological enhancement in addition to the physical mode of action. Thus, in vitro studies are strongly needed to characterize the effect of AuNPs, including cell toxicity, uptake dynamics, radiation-induced cell survival, etc.

Promising results have been obtained with AuNPs plus RT using kV X-rays. However, low-energy photon beams are not suitable for external RT of deep-seated tumors. The greater penetration power of MV photon beams makes this energy range preferable for clinical RT of most pathologies. Given the treatment units available in RT departments, the in vitro radiosensitizer effect of AuNPs with MV beams has been investigated on various human tumor cell lines including breast, brain, colon, lung, and prostate [16]. Tudda et al. tested the irradiation of the MDA-MB-231 breast tumor cell line with 6 MV in the presence of 15 nm AuNPs. A Dose Enhancement Factor (DEF) of 1.14 ± 0.06 and 1.33 ± 0.06 was obtained for MV and kV X-rays, respectively [17]. For the same cell line, Jain et al. compared cell survival fractions after irradiation with 160 kV, 6 MV, and 15 MV X-ray beams using 1.9 nm AuNPs. Sensitizer Enhancement Ratios (SER) of 1.41, 1.29, and 1.16 were obtained, respectively [18]. For the breast cancer cell line MCF-7 and using glucose-bound AuNPs of 16 nm, Soleymanifard et al. obtained smaller gains after irradiation with 6 MV beams than with kV as quantified by the inhibition of cell proliferation (39% vs. 64%, respectively) [19]. Using the HeLa cell line, Chithrani et al. tested the effects of AuNPs of different sizes and different beam energies. Photon beams of 105 kV and 50 nm AuNPs was the combination that showed the highest Radiosensitization Enhancement Factor (REF) of 1.66. This value was reduced to 1.17 when 6 MV photon beams were used [20]. These findings are consistent with those obtained for the MDA-MB-231 cell line incubated with 49 nm and 16 nm thioglucose-bound AuNP and irradiated with 6 MV beams. A significantly larger radiosensitization was obtained with 49 nm AuNPs than with 16 nm (1.86 vs. 1.49, respectively, *p* < 0.005) [21]. For other cell lines, a gain in irradiation in the presence of AuNPs, either expressed as DEF, SER, or REF, of up to 1.74 was obtained with different types of AuNPs combined with clinical beams [22,23,24,25,26,27].

For pancreatic cancer, the in vitro and in vivo potential of AuNPs for RT using low-energy photon beams has been assessed [28]. In this study, KPC and PANC-1 cells were incubated with poly(l-glutamic acid-co-l-lysine), or P(Glu-co-Lys)1:5, gold nanorods and irradiated with 250 kV X-rays under extracellular tumor acidosis and tumor microenvironment hypoxia. A DEF_10%_ of 1.24 and 1.1 was obtained for KPC and PANC-1 cells at a pH of 6.5, respectively. Interestingly, a similar radiosensitization was obtained for both cell lines under hypoxia conditions. The radiosensitization of pancreatic cancer in vivo was also evaluated in a subcutaneous tumor model performed in male mice using KPC cells. After the establishment of tumor mass, animals were irradiated 24 h after intravenous administration of P(Glu-co-Lys)1:5 or PEGylated gold nanorods. The combination of RT plus P(Glu-co-Lys)1:5 gold nanorods showed the best treatment efficacy, compared to RT alone or RT plus PEGylated-gold nanorods, even in the presence of extensive hypoxic tumor regions.

Still, for pancreatic cancer, Brero et al. tested 19.2 nm magnetic NPs (Fe_3_O_4_) in BxPC-3 cells that were irradiated with 6 MV photons, and concluded that these NPs at 50 μg/mL conferred a 50–60% additive effect on cell death compared to RT alone [29]. Detappe et al. evaluated 3.5 nm gadolinium-based NPs during irradiation with 220 kV of PANC-1 [30]. They concluded that 0.43 mg/mL of these NPs led to a SER of 1.37. Enhanced DNA damage and higher apoptosis were obtained in irradiated groups previously incubated with NPs compared to RT alone. Finally, Yoshida et al. assessed the radiosensitization effect of 190 nm AuNP microgels in mice injected with MIAPaCa-2 human pancreatic cancer cells and concluded that tumor growth was effectively suppressed in mice injected with AuNP microgels when 150 kV X-ray irradiation was performed [31].

Motivated by the challenges and difficulties found in pancreatic cancer therapy, this study reports an exploratory work involving the in vitro assessment of the radiation-sensitizing potential of target-specific AuNPs during the RT treatment of human pancreatic adenocarcinoma cells, BxPC-3, with MV X-rays. For this purpose, AuNPs coated with hyaluronic acid and oleic acid (HAOA-AuNPs) or with a BBN coating (BBN-AuNPs) were used. The impact of these AuNP formulations together with RT was assessed by viability assays.

## 2. Materials and Methods

### 2.1. Synthesis of HAOA-AuNPs and BBN-AuNPs

AuNPs were prepared according to previous methods [10,32,33] and HAOA-AuNPs according to [8]. Gold (III) chloride trihydrate (HauCl_4_‧3H_2_O), silver nitrate (AgNO_3_), L-ascorbic acid (L-AA), rosmarinic acid (RA), hyaluronic acid (HA) from *Streptococcus equi* (MW~1.5–1.8 × 10^6^ Da), and oleic acid (OA; MW = 282.46 g/mol) were supplied from Sigma-Aldrich (Steinheim, Germany). Briefly, fresh solutions of gold salt, silver nitrate, L-ascorbic acid, and rosmarinic acid were prepared [8,10,32,33]. The reaction of preparing uncoated AuNPs was carried out at 800 rpm for 15 min in a stirring plate (Fisherbrand ARE Hotplate Stirrer, Bradford, UK). The coating was previously prepared by mixing HA and OA, followed by stirring the suspension overnight at 400 rpm at 60 °C. On the next day, the coating suspension (HAOA) was added to the AuNP cores (proportion 1:1, *v*/*v*) and stirred for 30 min at 800 rpm at room temperature. Finally, HAOA-AuNPs were centrifuged (7200× *g* for 15 min) stored at 4 °C and protected from the light [32].

BBN-AuNPs were prepared as described in Silva et al. [34]. Firstly, AuNPs stabilized with 2-[4,7-bis(carboxymethyl)-10-[2-(3-sulfanylpropanoylamino) ethyl]-1,4,7,10-tetrazacyclododec-1-yl] acetic acid (TDOTA) were synthesized. The final solution was centrifuged at 1000 rpm for 20 min and the pellet obtained was washed two times with methanol (MeOH) and two times with H_2_O. NPs were then dried at reduced pressure. Thioctic acid terminated bombesin peptide (SS-BBN) was prepared in an automated peptide synthesizer. The final product was prepared by mixing AuNP-TDOTA with the thiolated peptide in a 1:2 ratio (*w/w*) at room temperature for 2 h. Briefly, 800 µL of MeOH was added to a 200 µL suspension of AuNP-TDOTA (5 mg/mL in deionized water) and then 1 mL of a solution of SS-BBN (2 mg, 1.77 µmol, in MeOH) was added. The mixtures were stirred at room temperature for 2 h. Then, centrifugation at 12,000 rpm for 5 min was performed. The BBN-AuNPs were washed with MeOH and H_2_O and lyophilized [34].

AuNPs were characterized in terms of morphology and mean particle size by AFM and by Dynamic Light Scattering (DLS) (by Number), respectively. For the mean size and polydispersity index (PdI), samples were diluted in water (1:10) and analyzed through DLS (Zetasizer Nano S; Malvern Instruments, Malvern, UK) in three series of 11 measurements of each analyzed sample. For AFM, 40 μL of each sample was placed on a freshly cleaved mica surface and allowed to dry for one hour before analysis. Images were acquired by Multimode 8 HR coupled to Nanoscope V Controller (Bruker, Coventry, UK), using a peak force tapping and ScanAssist mode. The tip model used was scanasyst-air 0.4 N/m, Bruker.

### 2.2. Cell Culture

Human pancreatic adenocarcinoma BxPC-3 (ATCC© CRL-1687TM, LGC Standards, Barcelona, Spain) cells were cultured in RPMI-1640, supplemented with 10% fetal bovine serum and 100 IU/mL of penicillin and 100 μg/mL streptomycin (Gibco, Thermo Fisher Scientific, Waltham, MA, USA), further designated as a complete medium. Cells were kept at 37 °C, under a 5% CO_2_ atmosphere. Maintenance of cultures was performed every two to three days until cells reached a confluence of about 80%.

Before irradiation, BxPC-3 cells were trypsinized and suspensions with 4 × 10^4^ cells/mL were prepared. Cells were seeded in 96-well plates (200 μL) and allowed to adhere overnight. Then, the medium was discarded, and cells were incubated with AuNPs for 4 h to allow internalization or with complete medium (control) [10,35,36]. After the incubation period, the supernatant was discarded and 100 μL of the complete medium was added to each well to proceed to irradiation.

### 2.3. Irradiation Setup

**Figure 1:**
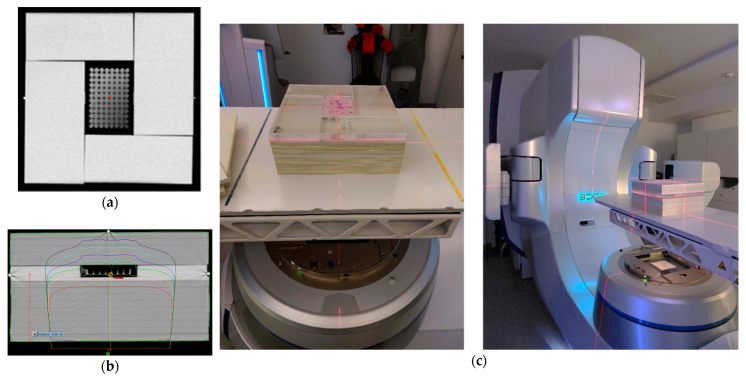
(**a**) Computer tomography (CT) image of the phantom and (**b**) radiation dose distribution used for irradiation of BxPC-3 cells obtained in the treatment planning system Eclipse. The isodoses show that all cells were irradiated with the same radiation dose; (**c**) a posterior 6 MV beam from a Varian Edge linear accelerator was used. Each 96-well plate was placed at the isocenter at a 10 cm depth of the phantom (indicated by the lasers).

All irradiations were performed on a Varian Edge medical linear accelerator (Varian Medical Systems, Inc., Palo Alto, CA, USA) from the Champalimaud Foundation. For irradiation, BxPC-3 cell plates were placed inside the phantom at a depth of 10 cm. The phantom was composed of a stack of polystyrene plates of 30 × 30 cm^2^; holding a set of PMMA plates mounted around the 96-well plate, designed to avoid the presence of air around the plate (Figure 1). Five-centimeter polystyrene plates were placed above the cell plates to allow backscatter. A Computer Tomography (CT) of this phantom was acquired in a Philips Brilliance Big Bore 16 Slice CT Simulator (Koninklijke Philips N.V., Eindhoven, The Netherlands). Treatment planning for dosimetry was made using the treatment planning system Eclipse (Eclipse Software v15.6, Varian Medical Systems, Inc., Palo Alto, CA, USA). Irradiation was performed with a 6 MV photon beam produced by a standard flattening filter, with a 20 × 20 cm^2^ field size at a dose rate of 600 MU/min with cells placed at the isocenter of the linear accelerator. A posterior beam was used to prevent the beam from having to pass through air before reaching the cells. Cells, in the absence and presence of AuNPs, were irradiated with doses ranging from 2 to 10 Gy.

### 2.4. Viability Assays

The viability of BxPC-3 cells after incubation and irradiation at pre-selected times was evaluated by the MTT (3-[4,5-dimethylthiazol-2-yl]-2,5 diphenyl tetrazolium bromide) assay. For MTT assays, initially, a protocol was established aiming to determine optimal irradiation conditions where different factors were tested [37]. To validate the best MTT assay conditions for the subsequent assays, the influence of post-irradiation time on cell viability was first assessed. Cells were thus irradiated with 2, 5, and 10 Gy and cell viability was evaluated by MTT assay 24, 48, and 72 h post-irradiation. No AuNPs were used during this assay.

After irradiation, 100 μL of complete medium was added to each well. Then, after 24, 48, or 72 h, the medium was discarded followed by washing with PBS twice, and 50 μL of MTT reagent at a concentration of 0.5 mg/mL in medium was added to each well. Following an incubation period of 2–4 h, 100 μL of dimethyl sulfoxide (DMSO) was added to each well to solubilize the formazan crystals. After solubilization, absorbance was measured at 570 nm in a microplate reader (BioTek ELx800; BioTek Instruments, Inc., Winooski, VT, USA) [36]. Cell plates transported to Champalimaud Foundation, but not irradiated nor incubated with AuNPs, were defined as the control group, corresponding to 100% cell viability. The results of the test groups (RT alone, AuNPs alone, and RT + AuNPs) were normalized to the control group.

To investigate the impact that AuNPs induced during RT, BxPC-3 cells were incubated with HAOA-AuNPs at different gold concentrations (50, 200, and 400 μM) and with BBN-AuNPs at 50 and 200 μM. Cells were irradiated using the described setup with 2.0, 3.5, and 5.0 Gy. After irradiation, cells were incubated for 48 and 72 h, and cell viability was evaluated by MTT assay.

### 2.5. Statistical Analysis

Cell viability measurements of BxPC-3 cells were presented as the mean ± standard deviation (SD). Data analysis was performed using GraphPad Prism version 9.4.0 (GraphPad Software, Inc., San Diego, CA, USA), and a *p*-value < 0.05 was considered statistically significant. Cell viabilities were compared by two-way ANOVA followed by Tukey’s multiple comparisons test.

The Coefficient of treatment Interaction (CI) was calculated using the expression

$$CI=\frac{{VC}_{RT+AuNP}}{{VC}_{RT}{VC}_{AuNP}}$$

where VC_RT+AuNP_ is the percentage of viable cells after the combined treatment, VC_RT_ is the percentage of viable cells after RT alone and VC_AuNP_ is the percentage of viable cells after treatment with the AuNPs alone. It was considered that, for CI values below one, there was a synergy between the two treatments while, for values equal to one or larger than one, the effect was additive or antagonistic, respectively [38].

## 3. Results

### 3.1. Characterization of HAOA-AuNPs and BBN-AuNPs

**Figure 2:**
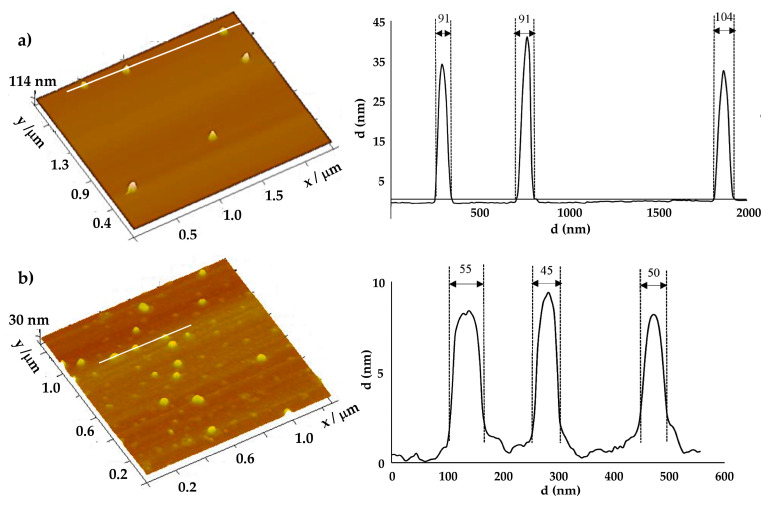
3D Atomic Force Microscopy images with corresponding cross-section profiles for the two AuNP formulations: (**a**) HAOA-AuNPs and (**b**) BBN-AuNPs.

AuNPs were evaluated in terms of their morphology by AFM and in terms of their mean particle size by AFM and DLS (Table 1). The AFM technique demonstrated that both AuNPs presented a spherical morphology (Figure 2) with a mean particle size of 83 ± 20 nm and 49 ± 12 nm for HAOA-AuNPs and BBN-AuNPs, respectively. By DLS, a mean particle size of 118 nm for HAOA-AuNPs (PdI < 0.3) was obtained while, for BBN-AuNPs, a mean particle size of 78.2 nm was reported by Silva et al. [39]. Within the range of uncertainties, the size of NPs obtained by AFM and DLS are consistent even though larger mean sizes were obtained by DLS.

### 3.2. Influence of Post-Irradiation Assessment Time

**Figure 3:**
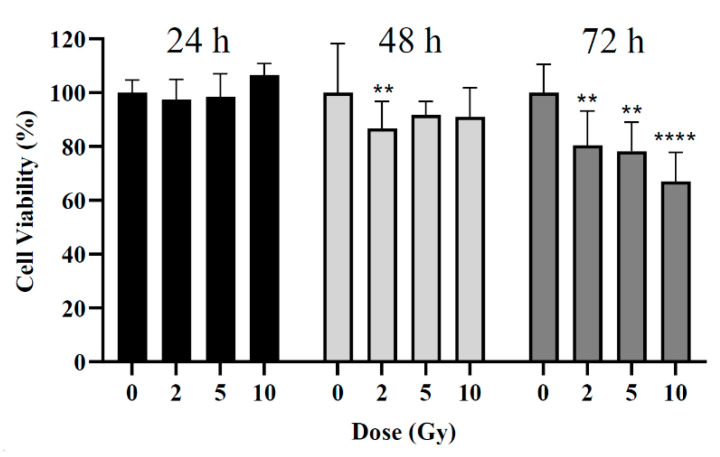
Cell viability of BxPC-3 cell line 24, 48, and 72 h after irradiation with doses ranging from 2 to 10 Gy (without AuNPs). Cells not irradiated were considered the control group. ** *p* < 0.01, **** *p* < 0.0001 compared to control at the same incubation time.

The influence of radiation dose on cell viability was assessed at 24, 48, and 72 h post-irradiation by MTT assay. Three different radiation doses were used: 2, 5, and 10 Gy. For the 24 h post-irradiation time, no significant differences were noted between the control (i.e., cells non-irradiated) and cells irradiated (Figure 3). For the 48 h post-irradiation time, a statistically significant difference was obtained only for cells irradiated with 2 Gy with a reduction in cell viability of 13 ± 10% compared to the control group (*p* = 0.002). Neither 5 Gy nor 10 Gy led to the expected larger loss in cell viability (*p* > 0.09). For the 72 h post-irradiation time, statistically significant reductions in cell viability of about 20 ± 13%, 22 ± 11%, and 33 ± 12% were obtained after irradiation with 2, 5, and 10 Gy, respectively (*p* = 0.004; *p* = 0.001; *p* < 0.0001).

### 3.3. Impact of AuNPs during RT

**Figure 4:**
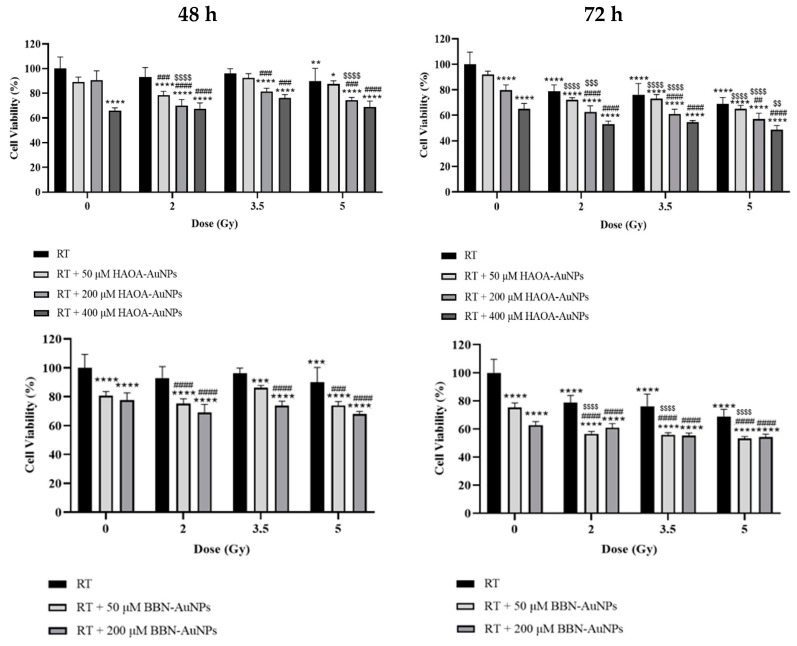
Cell viability of BxPC-3 cell line 48 and 72 h after photon beam irradiation, with radiation doses ranging from 2 to 5 Gy, and with HAOA-AuNPs and BBN-AuNPs with concentrations ranging from 50 to 400 μM compared to non-treated cells (0 Gy). * *p* < 0.05, ** *p* < 0.01, *** *p* < 0.001, and **** *p* < 0.0001 compared to control, i.e., non-treated cells. ## *p* < 0.01, ### *p* < 0.001, and #### *p* < 0.0001 compared to RT alone at the same radiation dose. $$ *p* < 0.01, $$$ *p* < 0.001, and $$$$ *p* < 0.0001 compared to AuNPs alone at the same concentration.

The potential effect of AuNPs during RT of BxPC-3 cancer cells was evaluated by MTT assay 48 and 72 h post-irradiation. Cell viability was assessed for HAOA-AuNPs at 50, 200, and 400 µM and BBN-AuNPs at 50 and 200 µM using radiation doses ranging from 2 to 5 Gy. For the 48 h post-irradiation time, a statistically significant reduction in cell viability was obtained with 400 μM of HAOA-AuNPs alone and 50 μM or 200 μM of BBN-AuNPs alone, compared to control cells (*p* < 0.0001, indicated by the stars in the left panels of Figure 4). Neither 50 μM nor 200 μM, of HAOA-AuNPs alone induced a significant difference in cell viability compared to the control (*p* = 0.055; *p* = 0.165, respectively). For RT alone, 5 Gy was required to significantly reduce cell viability compared to the control group (*p* = 0.001). For the 48 h post-irradiation time, the combined treatment of 2 Gy + 50 μM of HAOA-AuNPs induced a reduction in cell viability of 22 ± 3% and 2 Gy + 200 μM a loss in cell viability of approximately 30 ± 5% compared to the control (*p* < 0.0001). However, the cytotoxic effect of the combined treatment with radiation doses of 3.5 and 5.0 Gy was not enhanced compared to that obtained at 2 Gy. Overall, a higher cell viability for higher radiation doses was obtained. This validates the above findings and therefore the need for longer post-irradiation times to evaluate the biological effects of RT.

The results of MTT assays performed 72 h post-irradiation are shown in Figure 4 (right panels). Within the range of radiation doses tested, RT alone reduced cell viability from 21 to 31 ± 5% (*p* < 0.0001). No significant difference in cell viability was obtained between 50 µM HAOA-AuNPs alone and the control (*p* = 0.26). On the other hand, a statistically significant reduction in cell viability of approximately 20 ± 4% and 35 ± 4% for HAOA-AuNPs alone at 200 and 400 µM, respectively, was obtained compared to control cells (*p* < 0.0001). Similarly, for cells incubated with BBN-AuNPs alone, a statistically significant loss in cell viability of 25 ± 3% and 37 ± 3% was obtained for 50 and 200 µM AuNPs, respectively, when compared with control cells (*p* < 0.0001).

**Figure 5:**
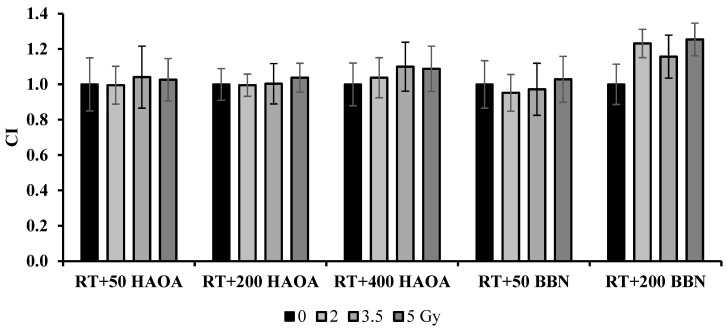
Results of the coefficient of treatment interaction (CI) for the NPs tested 72 h post-irradiation. It was considered that there was a synergy between the two treatments when CI < 1 while, for CI = 1, the effect was considered additive and, for CI > 1, the effect was considered antagonistic [38]. Doses of radiation from 2 to 5 Gy were delivered with concentrations of AuNP of 50 to 400 μM of HAOA and BBN-AuNP.

The combined treatment of RT with HAOA-AuNPs at 50, 200, and 400 µM induced a loss in cell viability of 28–35%, 37–43%, and 45%-51%, respectively, compared to the control (*p* < 0.0001). For RT + HAOA-AuNPs, an almost linear relation was obtained between cell viability and concentration of HAOA-AuNPs for the combined treatment (Figure S1—Supplementary Materials). Furthermore, statistically significant reductions in cell viability were obtained between RT with HAOA-AuNPs (200 µM and 400 µM) and RT alone for the same radiation dose (comparison represented by the cardinal symbols in Figure 4), but not for 50 µM of AuNPs (*p* > 0.6). Also, a statistically significant reduction in cell viability was obtained between RT + HAOA-AuNPs at 50 and 200 μM and AuNPs alone at the same concentration (indicated by the dollar signs in Figure 4). Finally, a statistically significant loss in cell viability was obtained when combining a radiation dose of 5 Gy with HAOA-AuNPs at 400 μM compared to the same nanoparticle concentration. For the tested AuNP concentrations, and within the range of estimated uncertainties, the effects caused by the combined treatment were mainly caused by an additive effect between RT and AuNPs, as demonstrated by the calculated CI value (Figure 5).

For the 72 h post-irradiation time, the combined treatment of RT with 50 µM of BBN-AuNP induced a maximum loss in cell viability of 47 ± 1% when compared to control (*p* < 0.0001) and 22% compared to BBN-AuNP alone for the same concentration (*p* < 0.0001). A similar loss in cell viability was obtained with RT plus BBN-AuNPs at 200 μM, but this difference was not significantly different from the group of cells incubated with these AuNPs alone for the same concentration (*p* > 0.3). For the lowest concentration of BBN-AuNP tested, the combined effects with irradiation presented an additive effect (Figure 5).

## 4. Discussion

In the last two decades, important advances in cytotoxic agents and RT techniques have occurred without a clinical translation into significant overall survival benefits for pancreatic cancer patients [3]. These difficulties are partly due to the generally late detection of this pathology, reflecting also the inability to deliver higher doses of RT locally without causing severe toxicity to nearby healthy tissues [40,41]. The ability to successfully improve local tumor response may lead to an extension of overall survival. As pancreatic tumor response is radiation dose-dependent [41], improving RT by allowing a local dose escalation while maintaining or reducing the dose in nearby organs at risk, could potentially result in a better treatment outcome.

In this study, the potential of RT in combination with AuNPs for the treatment of locally advanced pancreatic cancer was investigated in vitro by the evaluation of the effects on cell viability via the MTT assay. This assay was used due to its multiple advantages: high reproducibility, rapid semi-automated reading, and comparatively low cost [42]. For MTT assays, preliminary studies were performed, aiming to determine optimal irradiation conditions. It was found that for treatments with RT alone, a 72 h post-irradiation time was the time point when significant changes in cell viability were measured. Masoudi-Khoram et al. obtained similar results after the irradiation of the human breast tumor cell line MDA-MB-231 where reductions in cell viability were only observed 72 h following irradiation with 6 and 10 Gy when compared to control [43]. For shorter periods, in general, cell viability increased with radiation dose, contradicting the expected biological effects of RT (Figure 3). These results are consistent with the findings from others. Kong et al. did not obtain the expected reduction in cell viability of the human breast cancer cell line (MCF-7) 48 h following irradiation with 10 Gy. Indeed, the authors reported an 11.5% increase in cell viability when compared to the control [44].

This increase in cell viability obtained for short post-irradiation periods with increasing radiation dose (Figure 3), might be related to the lag period of cells after irradiation. This delay is probably due to the time frame of cell death following irradiation, reflecting a damage repair that is cell-line dependent [42]. If cell viability is measured during the lag phase (i.e., too early), results are overestimated. For BxPC-3 cells, used in the present work, cell doubling time is 48 h to 60 h [45]. Thus, for incubation times lower than this period, cell viability may be overestimated. Additionally, the lag period is also influenced by the dose of radiation. Increasing the dose leads to an increased lag period and, consequently, it is necessary to consider the longest post-irradiation time points to accurately observe the losses in cell viability [42]. However, the feasibility of increasing post-irradiation time is limited by the possible overgrowth of the cells in the microplate, which is quite likely to occur for long incubation times. Thus, in this study, as the highest difference in cell viability between irradiated and control groups was observed for the 72 h post-irradiation time, this experimental condition was selected as the most appropriate for further assays.

Within the range of tested conditions, single treatment, either by RT or AuNPs, led to a maximum loss in cell viability of 31 ± 5% and 37 ± 4%, respectively, compared to the control. Aiming to assess the advantages of concomitant RT with AuNPs, pancreatic cancer cells were irradiated after 4 h of incubation with NPs. RT plus AuNPs induced a maximum loss in cell viability of 47 ± 1% with BBN-AuNP at 50 μM and of 51 ± 3% for RT plus HAOA-AuNP at 400 μM. For HAOA-AuNPs, a linear relationship between concentration and cell viability was obtained (Figure S1—Supplementary Materials). Higher concentrations of HAOA-AuNPs concomitantly delivered with RT could potentially result in even better outcomes, provided these concentrations could be safely delivered to patients.

For BxPC-3 cells irradiated with MV beams, without AuNPs, cell survival fraction at 2 Gy, SF2, takes values of about 60%, and SF5 is around 20% [46]. In this study, for a radiation dose of 2 and 5 Gy, cell viability normalized to the control was 79 ± 5% and 69 ± 5%, respectively, while RT plus AuNPs reduced cell viability by an additional 15–26%. Discrepancies between the results obtained with MTT and clonogenic assays have been previously reported [47,48] and, for RT in the presence of AuNPs, these discrepancies could be even more accentuated as there could be more DNA damage to be repaired. The CI indicates additive effects for most tested cases (Figure 5), but the results obtained in this study may underestimate the true outcome of the combined treatment. The interaction between RT and the tested AuNPs on cell survival needs to be thoroughly investigated.

The morphology of the NPs has a strong influence on their radiosensitizing capabilities. AFM has shown that both AuNPs used in this study are spherical in shape, facilitating internalization into tumor cells [15,20]. Ma et al. evaluated the radiosensitization effect in RT of three types of gold nanostructures: AuNPs with a spherical shape, gold nanorods, and gold nanospikes. Radiosensitization was related to the ability of cellular internalization, which was more efficient for spherical AuNPs, followed by gold nanospikes, and then gold nanorods (SER of 1.62, 1.37, and 1.21, respectively) [48].

AuNP size evaluated by AFM was smaller compared to DLS (Table 1). This difference arises from the fundamental principles of these techniques [49]. DLS measures the hydrodynamic diameter of particles in suspension, capturing their behavior in a liquid medium. Thus, the measured diameter includes the core of the NPs plus any surrounding solvent molecules, polymer coatings, or adsorbed molecules that move with the particle. AFM measures the physical size of the particle without the surrounding solvent layer, measuring particles in a dry state on a substrate, after cleaning, purification, and recovery of that suspension, which might cause some changes in particle size due to drying effects or particle–substrate interactions. Furthermore, AFM can distinguish between individual particles and aggregates, providing more accurate measurements of single particles. In contrast, DLS provides the average size of a large number of particles in suspension. DLS is thus sensitive to particle aggregation, which can lead to larger size readings if particles clump together. Therefore, while both techniques provide valuable information, their results should be interpreted within the context of their measurement principles, and differences in reported sizes are to be expected.

The outcome for RT plus BBN-AuNPs at a low concentration and combined treatment using HAOA-AuNPs at a high concentration was similar, indicating the influence of the size and/or coating of the NPs on the RT effect and, ultimately, on the potential of the combined treatment of RT with AuNPs. Because HAOA-AuNPs and BBN-AuNPs are substantially different both in size and in coating, our results cannot predict which structural feature contributed more to the observed biological effect. The morphology, the nature of the coating, and the particle size may interfere with the level of cellular internalization, cytotoxicity, and stability, among other factors. Successful nanoparticle internalization into target cells and proximity to the cell nucleus is paramount to achieving the desired tumor-cell kill effect for which nanoparticle size is one of the key factors [15]. The presence of high atomic number NPs in tumor cells during RT mostly promotes the photoelectric effect, enhancing the number of Auger electrons released in the vicinity of AuNPs. While secondary electrons in the form of photoelectrons can travel up to a distance of several cells, low-energy Auger electrons have ranges of less than 10 nm in tissues. Consequently, these electrons offer a very precise and local dose deposition inside cells contributing to cell death while avoiding radiosensitization in nearby tissues [50]. For therapy, nanoparticles should primarily accumulate in the tumor and the distribution to non-target tissues should be minimized. However, some degree of diffusion or systemic distribution may occur depending on the specific characteristics of the nanoparticles and the tumor environment. Small nanoparticles (<20 nm) show better tumor penetration capabilities and diffuse more uniformly within tumor tissues. However, they can be easily pumped back into the bloodstream and rapidly expelled from the body, resulting in insufficient tumor accumulation [51,52]. But particle size also influences the NP biodistribution. De Jong et al. assessed AuNP concentration in different organs after intravenous injection of male rats with spherical AuNPs ranging from 10 to 250 nm [53]. For 10 nm AuNPs, the percentage of gold in the kidneys, brain, reproductive organs, thymus, and heart was much higher than for 50 to 250 nm AuNPs while 100 and 250 nm particles were not detected in the lung [53]. Larger AuNPs are advantageous due to their low toxicity, higher tumor retention, and stability, which facilitate functionalization [9]. But AuNP size and coating strongly impact the number of Auger electrons that can effectively escape from the nanoparticle as many can be stopped inside the nanoparticle itself [7,54]. Efforts are continually being made to optimize nanoparticle design and delivery strategies to enhance their tumor-targeting capabilities while minimizing off-target effects.

This exploratory work aimed to perform an initial assessment of the potential of concomitant RT with two targeted-specific AuNPs. NP stability, safety, and biocompatibility of HAOA and BBN-AuNPs were already addressed in vitro and in vivo [8,14,33,34,35,39,55]. For concomitant RT with AuNPs differing in size, coating, charge, etc., a deeper understanding of the biological mechanisms responsible for cell killing is needed. Moreover, the distribution of AuNPs in tumor and normal tissues must be assessed. During irradiation, NPs should preferentially accumulate in the tumor to maximize the local radiation boost in malignant cells. In that case, the response of normal tissues to RT + AuNPs will be similar to those obtained with current RT techniques as organs-at-risk sparing is accomplished by the conformity of the 3-dimensional radiation dose distribution, while appropriate NP functionalization will promote its accumulation only in target cells. Innovative radiation dose calculation algorithms considering the addition of AuNPs are needed to accurately quantify the administered radiation dose. Finally, a deeper understanding of the interaction of AuNPs with the radiation field with increasing depth will have to be thoroughly investigated [56]. Before clinical translation, additional research, aiming to address these key points using in vitro and in vivo assays together with Monte Carlo simulations, is deemed necessary.

## 5. Conclusions

The present study consisted of an exploratory analysis of the effect of AuNPs of different sizes and coatings, HAOA-AuNPs and BBN-AuNPs, on human pancreatic adenocarcinoma BxPC-3 cells irradiated with a clinical 6 MV photon beam. Cell viability assays showed that the effect of RT alone on BxPC-3 cells seems to be negligible for short post-irradiation times. The 72 h post-irradiation time proved to be more adequate to evaluate the effects of the combined treatment because it implied more significant differences in the cell viability values after treatment with low doses of RT and AuNP.

For the 72 h post-irradiation time, the combined treatment of RT either with HAOA-AuNPs at 200 μM or with BBN-AuNPs at 50 μM led to a mean cell viability reduction of around 45% compared to no treatment. In addition, the effect of the combined treatment seems to have originated from an additive effect caused by RT and AuNPs. Thus, there appears to be a potential benefit from the combined treatment for both formulations of AuNPs.

## Figures and Tables

**Table 1:** Mean particle size of HAOA-AuNPs and BBN-AuNPs. Data are presented as mean value ± SD.

	Mean Size (nm)		
AuNP Formulation	AFM	DLS	PdI
HAOA-AuNPs	83 ± 20	118 ± 7	0.20 ± 0.01
BBN-AuNPs	49 ± 12	78 (Ref. [39])	0.89 (Ref. [39])

Abbreviations: HAOA-AuNPs = gold nanoparticles coated with hyaluronic and oleic acids; BBN-AuNPs = gold nanoparticles coated with bombesin; DLS = Dynamic Light Scattering; PdI = polydispersity index; AFM = Atomic Force Microscopy.

## Data Availability

Data will be made available upon reasonable request.

## Supplementary Materials

The following supporting information can be downloaded at: https://www.mdpi.com/article/10.3390/pharmaceutics16070900/s1, Figure S1: Cell viability with increasing AuNP concentration for the HAOA-AuNP (open symbols) and BBN-AuNP (closed symbols) for different doses of radiation. Linear fit was applied to HAOA-AuNP data and a R2-value higher than 0.96 was obtained.

## References

[1] Sung H., Ferlay J., Siegel R.L., Laversanne M., Soerjomataram I., Jemal A., Bray F. (2021). Global Cancer Statistics 2020: GLOBOCAN Estimates of Incidence and Mortality Worldwide for 36 Cancers in 185 Countries. CA Cancer J. Clin..

[2] Rawla P., Sunkara T., Gaduputi V. (2019). Epidemiology of Pancreatic Cancer: Global Trends, Etiology and Risk Factors. World J. Oncol..

[3] McGuigan A., Kelly P., Turkington R.C., Jones C., Coleman H.G., McCain R.S. (2018). Pancreatic Cancer: A Review of Clinical Diagnosis, Epidemiology, Treatment and Outcomes. World J. Gastroenterol..

[4] Kuncic Z., Lacombe S. (2018). Nanoparticle Radio-Enhancement: Principles, Progress and Application to Cancer Treatment. Phys. Med. Biol..

[5] Her S., Jaffray D.A., Allen C. (2017). Gold Nanoparticles for Applications in Cancer Radiotherapy: Mechanisms and Recent Advancements. Adv. Drug Deliv. Rev..

[6] Bromma K., Chithrani D.B. (2020). Advances in Gold Nanoparticle-Based Combined Cancer Therapy. Nanomaterials.

[7] Schuemann J., Berbeco R., Chithrani B.D., Cho S., Kumar R., Mcmahon S., Sridhar S., Krishnan S. (2016). Roadmap to Clinical Use of Gold Nanoparticles for Radiosensitization. Int. J. Radiat. Oncol. Biol. Phys..

[8] Lopes J., Ferreira-Gonçalves T., Ascensão L., Viana A.S., Carvalho L., Catarino J., Faísca P., Oliva A., de Barros D.P.C., Rodrigues C.M.P. (2023). Safety of Gold Nanoparticles: From In Vitro to In Vivo Testing Array Checklist. Pharmaceutics.

[9] Ferreira-Gonçalves T., Ferreira D., Ferreira H.A., Reis C.P. (2021). Nanogold-Based Materials in Medicine: From Their Origins to Their Future. Nanomedicine.

[10] Silva C.O., Rijo P., Molpeceres J., Ascensão L., Roberto A., Fernandes A.S., Gomes R., Coelho J.M.P., Gabriel A., Vieira P. (2016). Bioproduction of Gold Nanoparticles for Photothermal Therapy. Ther. Deliv..

[11] Amaral M., Charmier A.J., Afonso R.A., Catarino J., Faísca P., Carvalho L., Ascensão L., Coelho J.M.P.P., Manuela Gaspar M., Reis C.P. (2021). Gold-Based Nanoplataform for the Treatment of Anaplastic Thyroid Carcinoma: A Step Forward. Cancers.

[12] Sakurai Y., Harashima H. (2019). Hyaluronan-Modified Nanoparticles for Tumor-Targeting. Expert Opin. Drug Deliv..

[13] Wei H.J., Yin T., Zhu Z., Shi P.F., Tian Y., Wang C.Y. (2011). Expression of CD44, CD24 and ESA in Pancreatic Adenocarcinoma Cell Lines Varies with Local Microenvironment. Hepatobiliary Pancreat. Dis. Int..

[14] Silva F., Campello M.P.C., Paulo A. (2021). Radiolabeled Gold Nanoparticles for Imaging and Therapy of Cancer. Materials.

[15] Chen Y., Yang J., Fu S., Wu J. (2020). Gold Nanoparticles as Radiosensitizers in Cancer Radiotherapy. Int. J. Nanomed..

[16] Rosa S., Connolly C., Schettino G., Butterworth K.T., Prise K.M. (2017). Biological Mechanisms of Gold Nanoparticle Radiosensitization. Cancer Nanotechnol..

[17] Tudda A., Donzelli E., Nicolini G., Semperboni S., Bossi M., Cavaletti G., Castriconi R., Mangili P., Del Vecchio A., Sarno A. (2021). Breast Radiotherapy with Kilovoltage Photons and Gold Nanoparticles as Radiosensitizer: An In Vitro Study. Med. Phys..

[18] Jain S., Coulter J.A., Hounsell A.R., Butterworth K.T., McMahon S.J., Hyland W.B., Muir M.F., Dickson G.R., Prise K.M., Currell F.J. (2011). Cell-Specific Radiosensitization by Gold Nanoparticles at Megavoltage Radiation Energies. Int. J. Radiat. Oncol. Biol. Phys..

[19] Soleymanifard S., Rostami A., Aledavood S.A., Matin M.M., Sazgarnia A. (2017). Increased Radiotoxicity in Two Cancerous Cell Lines Irradiated by Low and High Energy Photons in the Presence of Thio-Glucose Bound Gold Nanoparticles. Int. J. Radiat. Biol..

[20] Chithrani D.B., Jelveh S., Jalali F., Van Prooijen M., Allen C., Bristow R.G., Hill R.P., Jaffray D.A. (2010). Gold Nanoparticles as Radiation Sensitizers in Cancer Therapy. Radiat. Res..

[21] Wang C., Jiang Y., Li X., Hu L. (2015). Thioglucose-Bound Gold Nanoparticles Increase the Radiosensitivity of a Triple-Negative Breast Cancer Cell Line (MDA-MB-231). Breast Cancer.

[22] Liu P., Jin H., Guo Z., Ma J., Zhao J., Li D., Wu H., Gu N. (2016). Silver Nanoparticles Outperform Gold Nanoparticles in Radiosensitizing U251 Cells in Vitro and in an Intracranial Mouse Model of Glioma. Int. J. Nanomed..

[23] Ahmad R., Schettino G., Royle G., Barry M., Pankhurst Q.A., Tillement O., Russell B., Ricketts K. (2020). Radiobiological Implications of Nanoparticles Following Radiation Treatment. Part. Part. Syst. Charact..

[24] Zhang X., Wang H., Coulter J.A., Yang R. (2018). Octaarginine-Modified Gold Nanoparticles Enhance the Radiosensitivity of Human Colorectal Cancer Cell Line Ls180 to Megavoltage Radiation. Int. J. Nanomed..

[25] Saberi A., Shahbazi-Gahrouei D., Abbasian M., Fesharaki M., Baharlouei A., Arab-Bafrani Z. (2017). Gold Nanoparticles in Combination with Megavoltage Radiation Energy Increased Radiosensitization and Apoptosis in Colon Cancer HT-29 Cells. Int. J. Radiat. Biol..

[26] Wolfe T., Chatterjee D., Lee J., Grant J.D., Bhattarai S., Tailor R., Goodrich G., Nicolucci P., Krishnan S. (2015). Targeted Gold Nanoparticles Enhance Sensitization of Prostate Tumors to Megavoltage Radiation Therapy In Vivo. Nanomedicine.

[27] Kazmi F., Vallis K.A., Vellayappan B.A., Bandla A., Yukun D., Carlisle R. (2020). Megavoltage Radiosensitization of Gold Nanoparticles on a Glioblastoma Cancer Cell Line Using a Clinical Platform. Int. J. Mol. Sci..

[28] Rauta P.R., Mackeyev Y., Sanders K., Kim J.B.K., Gonzalez V.V., Zahra Y., Shohayeb M.A., Abousaida B., Vijay G.V., Tezcan O. (2022). Pancreatic Tumor Microenvironmental Acidosis and Hypoxia Transform Gold Nanorods into Cell-Penetrant Particles for Potent Radiosensitization. Sci. Adv..

[29] Brero F., Albino M., Antoccia A., Arosio P., Avolio M., Berardinelli F., Bettega D., Calzolari P., Ciocca M., Corti M. (2020). Hadron Therapy, Magnetic Nanoparticles and Hyperthermia: A Promising Combined Tool for Pancreatic Cancer Treatment. Nanomaterials.

[30] Detappe A., Kunjachan S., Sancey L., Motto-Ros V., Biancur D., Drane P., Guieze R., Makrigiorgos G.M., Tillement O., Langer R. (2016). Advanced Multimodal Nanoparticles Delay Tumor Progression with Clinical Radiation Therapy. J. Control. Release.

[31] Yoshida A., Kitayama Y., Kiguchi K., Yamada T., Akasaka H., Sasaki R., Takeuchi T. (2019). Gold Nanoparticle-Incorporated Molecularly Imprinted Microgels as Radiation Sensitizers in Pancreatic Cancer. ACS Appl. Bio Mater..

[32] Lopes J., Miguel J., Coelho P., Manuel P., Vieira C., Silveira Viana A., Gaspar M.M., Reis C. (2020). Preliminary Assays towards Melanoma Cells Using Phototherapy with Gold-Based Nanomaterials. Nanomaterials.

[33] Ferreira-Gonçalves T., Nunes D., Fortunato E., Martins R., de Almeida A.P., Carvalho L., Ferreira D., Catarino J., Faísca P., Ferreira H.A. (2024). Rational Approach to Design Gold Nanoparticles for Photothermal Therapy: The Effect of Gold Salt on Physicochemical, Optical and Biological Properties. Int. J. Pharm..

[34] Silva F., Zambre A., Campello M.P.C., Gano L., Santos I., Ferraria A.M., Ferreira M.J., Singh A., Upendran A., Paulo A. (2016). Interrogating the Role of Receptor-Mediated Mechanisms: Biological Fate of Peptide-Functionalized Radiolabeled Gold Nanoparticles in Tumor Mice. Bioconjug. Chem..

[35] Ferreira-Gonçalves T., Gaspar M.M., Coelho J.M.P., Marques V., Viana A.S., Ascensão L., Carvalho L., Rodrigues C.M.P., Ferreira H.A., Ferreira D. (2022). The Role of Rosmarinic Acid on the Bioproduction of Gold Nanoparticles as Part of a Photothermal Approach for Breast Cancer Treatment. Biomolecules.

[36] Pinho S., Ferreira-Gonçalves T., Lopes J., Amaral M.N., Viana A.S., Coelho J.M.P., Gaspar M.M., Reis C.P. (2024). A Step Forward for the Treatment of Localized Prostate Cancer Using Gold Nanoparticles Combined with Laser Irradiation. Int. J. Mol. Sci..

[37] Martins A. (2022). Investigation of the Potential of Concomitant Radiation Therapy with Gold Nanoparticles for Pancreatic Cancer. Master’s Thesis.

[38] Singh H., Rana P.S., Singh U. (2019). Prediction of Drug Synergy Score Using Ensemble Based Differential Evolution. IET Syst. Biol..

[39] Silva F., Mendes C., D’onofrio A., Campello M.P.C., Marques F., Pinheiro T., Gonçalves K., Figueiredo S., Gano L., Ravera M. (2023). Image-Guided Nanodelivery of Pt(IV) Prodrugs to GRP-Receptor Positive Tumors. Nanotheranostics.

[40] Fiore M., Ramella S., Valeri S., Caputo D., Floreno B., Trecca P., Trodella L.E., Trodella L., Maria D’angelillo R., Coppola R. (2017). Phase II Study of Induction Chemotherapy Followed by Chemoradiotherapy in Patients with Borderline Resectable and Unresectable Locally Advanced Pancreatic Cancer OPEN. Sci. Rep..

[41] Petrelli F., Comito T., Ghidini A., Torri V., Scorsetti M., Barni S. (2017). Stereotactic Body Radiation Therapy for Locally Advanced Pancreatic Cancer: A Systematic Review and Pooled Analysis of 19 Trials. Int. J. Radiat. Oncol..

[42] Price P., McMillan T.J. (1990). Use of the Tetrazolium Assay in Measuring the Response of Human Tumor Cells to Ionizing Radiation. Cancer Res..

[43] Masoudi-Khoram N., Abdolmaleki P., Hosseinkhan N., Nikoofar A., Mowla S.J., Monfared H., Baldassarre G. (2020). Differential MiRNAs Expression Pattern of Irradiated Breast Cancer Cell Lines Is Correlated with Radiation Sensitivity. Sci. Rep..

[44] Kong T., Zeng J., Wang X., Yang X., Yang J., McQuarrie S., McEwan A., Roa W., Chen J., Xing J.Z. (2008). Enhancement of Radiation Cytotoxicity in Breast-Cancer Cells by Localized Attachment of Gold Nanoparticles. Small.

[45] Tan M.H., Nowak N.J., Loor R., Ochi H., Sandberg A.A., Lopez C., Pickren J.W., Berjian R., Douglass H.O., Chu T.M. (1986). Characterization of a New Primary Human Pancreatic Tumor Line. Cancer Investig..

[46] Wang P., Zhang J., Zhang L., Zhu Z., Fan J., Chen L., Zhuang L., Luo J., Chen H., Liu L. (2013). MicroRNA 23b Regulates Autophagy Associated with Radioresistance of Pancreatic Cancer Cells. Gastroenterology.

[47] Roa W., Zhang X., Guo L., Shaw A., Hu X., Xiong Y., Gulavita S., Patel S., Sun X., Chen J. (2009). Gold Nanoparticle Sensitize Radiotherapy of Prostate Cancer Cells by Regulation of the Cell Cycle. Nanotechnology.

[48] Ma N., Wu F.G., Zhang X., Jiang Y.W., Jia H.R., Wang H.Y., Li Y.H., Liu P., Gu N., Chen Z. (2017). Shape-Dependent Radiosensitization Effect of Gold Nanostructures in Cancer Radiotherapy: Comparison of Gold Nanoparticles, Nanospikes, and Nanorods. ACS Appl. Mater. Interfaces.

[49] Filippov S.K., Khusnutdinov R., Murmiliuk A., Inam W., Zakharova L.Y., Zhang H., Khutoryanskiy V.V. (2023). Dynamic Light Scattering and Transmission Electron Microscopy in Drug Delivery: A Roadmap for Correct Characterization of Nanoparticles and Interpretation of Results. Mater. Horiz..

[50] Retif P., Pinel S., Toussaint M., Frochot C., Chouikrat R., Bastogne T., Barberi-Heyob M. (2015). Nanoparticles for Radiation Therapy Enhancement: The Key Parameters. Theranostics.

[51] Danhier F. (2016). To Exploit the Tumor Microenvironment: Since the EPR Effect Fails in the Clinic, What Is the Future of Nanomedicine?. J. Control. Release.

[52] Tang L., Yang X., Yin Q., Cai K., Wang H., Chaudhury I., Yao C., Zhou Q., Kwon M., Hartman J.A. (2014). Investigating the Optimal Size of Anticancer Nanomedicine. Proc. Natl. Acad. Sci. USA.

[53] De Jong W.H., Hagens W.I., Krystek P., Burger M.C., Sips A.J.A.M., Geertsma R.E. (2008). Particle Size-Dependent Organ Distribution of Gold Nanoparticles after Intravenous Administration. Biomaterials.

[54] Peukert D., Kempson I., Douglass M., Bezak E. (2020). Gold Nanoparticle Enhanced Proton Therapy: A Monte Carlo Simulation of the Effects of Proton Energy, Nanoparticle Size, Coating Material, and Coating Thickness on Dose and Radiolysis Yield. Med. Phys..

[55] Lopes J., Ferreira-Gonçalves T., Figueiredo I.V., Rodrigues C.M.P., Ferreira H., Ferreira D., Viana A.S., Faísca P., Gaspar M.M., Coelho J.M.P. (2021). Proof-of-Concept Study of Multifunctional Hybrid Nanoparticle System Combined with NIR Laser Irradiation for the Treatment of Melanoma. Biomolecules.

[56] Schmidt R.M., Hara D., Vega J.D., Abuhaija M.B., Tao W., Dogan N., Pollack A., Ford J.C., Shi J. (2022). Quantifying Radiosensitization of PSMA-Targeted Gold Nanoparticles on Prostate Cancer Cells at Megavoltage Radiation Energies by Monte Carlo Simulation and Local Effect Model. Pharmaceutics.

