# Supplementary material for: Enhanced Cytotoxicity against a Pancreatic Cancer Cell Line Combining Radiation and Gold Nanoparticles
Source: Pharmaceutics. 2024 Jul 5;16(7):900. doi: 10.3390/pharmaceutics16070900 (PMC11280324; doi:10.3390/pharmaceutics16070900)
Supplement: Supplementary file 1 [file pharmaceutics-16-00900-s001.zip › pharmaceutics-3068910-supplementary.pdf]

pharmaceutics

MDPI

Article

Enhanced Cytotoxicity against a Pancreatic Cancer Cell Line combining Radiation and Gold Nanoparticles

Supplementary material

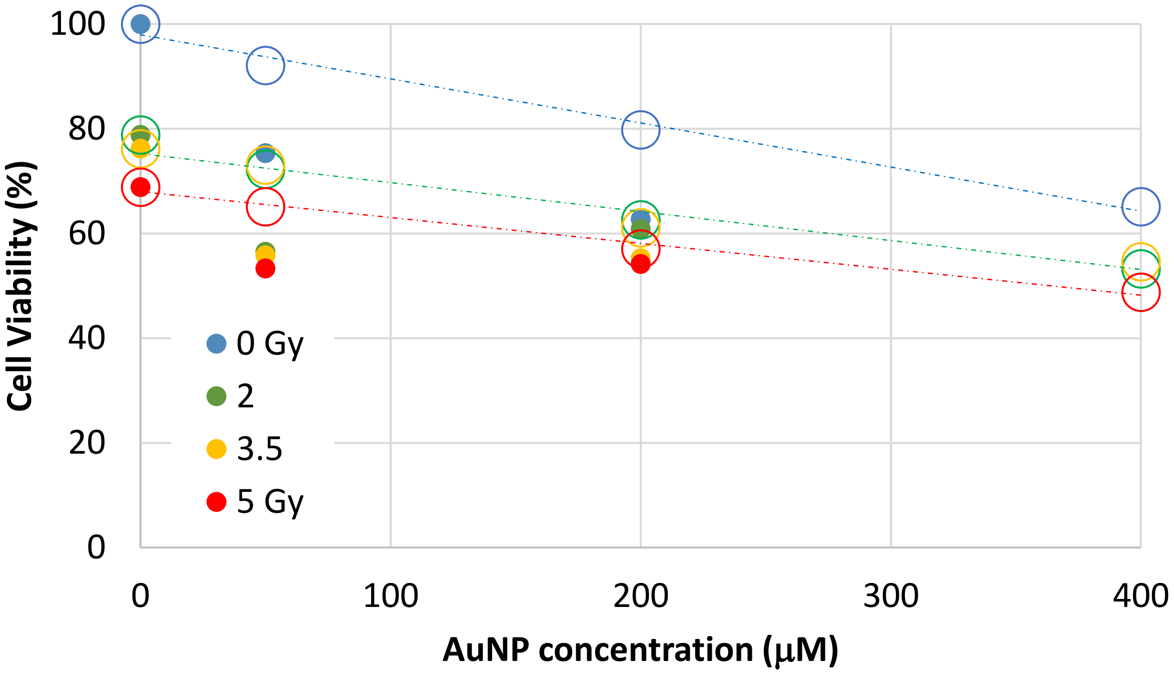

Figure S1. Cell viability with increasing AuNP concentration for the HAOA-AuNP (open symbols) and BBN-AuNP (closed symbols) for different doses of radiation. Linear fit was applied to HAOA-AuNP data and a R^2-value higher than 0.96 was obtained.
